# *Ceratothoa oestroides* Infection in European Sea Bass: Revealing a Long Misunderstood Relationship

**DOI:** 10.3389/fimmu.2021.645607

**Published:** 2021-03-11

**Authors:** M. Carla Piazzon, Ivona Mladineo, Ron P. Dirks, Elena Santidrián Yebra-Pimentel, Jerko Hrabar, Ariadna Sitjà-Bobadilla

**Affiliations:** ^1^Fish Pathology Group, Institute of Aquaculture Torre de la Sal - Consejo Superior de Investigaciones Científicas (IATS-CSIC), Castellón, Spain; ^2^Laboratory for Aquaculture, Institute of Oceanography and Fisheries, Split, Croatia; ^3^Biology Centre of the Czech Academy of Sciences, Institute of Parasitology, Ceske Budejovice, Czechia; ^4^Future Genomics Technology, Leiden, Netherlands

**Keywords:** aquaculture, *Ceratothoa oestroides*, cymothoidea, isopoda, *Dicentrarchus labrax*, RNA-seq, host-parasite interactions, immunoglobulin

## Abstract

*Ceratothoa oestroides* (Cymothoidea, Isopoda) is a generalist crustacean parasite that negatively affects the economic sustainability of European sea bass (*Dicentrarchus labrax*) aquaculture in the North-East Mediterranean. While mortalities are observed in fry and fingerlings, infection in juvenile and adult fish result in approximately 20% growth delay. A transcriptomic analysis (PCR array, RNA-Seq) was performed on organs (tongue, spleen, head kidney, and liver) from infected vs. *Ceratothoa*-free sea bass fingerlings. Activation of local and systemic immune responses was detected, particularly in the spleen, characterized by the upregulation of cytokines (also in the tongue), a general reshaping of the immunoglobulin (Ig) response and suppression of T-cell mediated responses. Interestingly, starvation and iron transport and metabolism genes were strongly downregulated, suggesting that the parasite feeding strategy is not likely hematophagous. The regulation of genes related to growth impairment and starvation supported the growth delay observed in infected animals. Most differentially expressed (DE) transcripts were exclusive of a specific organ; however, only in the tongue, the difference between infected and uninfected fish was significant. At the attachment/feeding site, the pathways involved in muscle contraction and intercellular junction were the most upregulated, whereas the pathways involved in fibrosis (extracellular matrix organization, collagen formation, and biosynthesis) were downregulated. These results suggest that parasite-inflicted damage is successfully mitigated by the host and characterized by regenerative processes that prevail over the reparative ones.

## Introduction

Fish parasites are increasingly being considered as an important economic drawback in aquaculture at a global scale (1). Among fish parasites, the crustaceans are the most diverse and ubiquitous group, encompassing some copepods that are considered as pests in salmon farming in both the Northern and Southern hemispheres. Caligid sea lice cause severe economic losses due to the reduced growth of infected fish, reduced marketability, and an increased cost of chemotherapy and mortality. Other crustaceans, such as the isopods, settle in the buccal cavity of fish, the gill chamber, or on the body surface, including the fins (2). Among them, Cymothoidea cause serious problems to fish kept in captivity. The damage caused by Cymothoidea resembles that of copepods, but the most marked effect of isopod infection is the destruction of the host tissues.

The current study focuses on the infection by the generalist crustacean, *Ceratothoa oestroides* (Cymothoidea, Isopoda), recognized as an important health issue in the North-East Mediterranean aquaculture of European sea bass (*Dicentrarchus labrax*), and to a lesser extent of gilthead sea bream (*Sparus aurata*) and of meager (*Argyrosomus regius*) (3–6). *Ceratothoa oestroides* parasitize the host buccal cavity, where paired growing individuals sexually differentiate and eventually reproduce, expelling hundreds of swimming infective stages (manca or pulli II) in the water column. The extent of the pathology is age-correlated. Therefore, the most susceptible category of fish suffering mortality after chronic emaciation are small fry or fingerlings, in which the damage results from abrupt cachexia and anemia (5). In contrast, larger fish show marginal lesions of the tongue, upper/lower jaws, and deformation of the buccal ventral part. Occasionally, severe ulcers and extensive granulomatous lesions with subsequent blindness can be observed (6, 7). Although the infected fish reach commercial size, the resulting 20% in growth reduction greatly influences the economic sustainability of the farming (8–10). Despite the economic impact caused by this parasite in aquaculture, the knowledge on host-parasite interactions is still limited. For example, whether the isopod is hematophagous or feeds upon the host tissues is still unknown. The exact feeding mechanisms cannot be fully elucidated by the appearance of the mouthparts (11, 12). A recent study observed tissue fragments around the mouthparts of the isopod and inside its mouth cavity, suggesting tissue grinding rather than blood sucking (13), as previously postulated (14).

The infection with *C. oestroides* has been characterized by a long-lasting and silenced inflammatory response that maintains the isopod at the attachment/feeding site with as minimal tissue damage as possible (13). It provokes no hemorrhages but causes a marked hyperplasia of the squamous epithelium on the tongue, accompanied by a weak-to-moderate inflammatory cell infiltration (macrophage-like cells, IgM^+^ cells, mast cells, and eosinophilic granulocytes) and a decrease in mucous cells counts. At the cellular level, in addition to the infiltration of some IgM^+^ cells (B lymphocytes, plasmablasts, and plasma cells), scarce cyclooxygenase 2 (Cox-2) activity accompanied by an elevated expression of the proliferating cell nuclear antigen (PCNA) was observed (13). The former is secreted by macrophages colonizing the oral mucosa to enhance the inflammatory response during the initial phase of infections through the conversion of arachidonic acid into prostaglandins (15), whereas PCNA is directly involved in DNA synthesis (16) and is a hallmark process of epithelial proliferation. Since uncontrolled proliferation can trigger apoptosis, as in the case of *Sparicotyle chrysophrii* (Monogenea, Polyopisthocotylea) and gill infection in gilthead sea bream (*S. aurata*) (17), it still remains to be determined whether this process also plays a role in sea bass-*Ceratothoa* interactions.

In view of the available microscopic and histopathological data associated with the infection with *C. oestroides* in European sea bass, the objective of the current work was to better understand the response of fingerlings, the most susceptible life-stage of sea bass to the isopod infection under natural conditions. This was done by exploring molecular changes at the parasite attachment/feeding site and at the systemic level by performing a transcriptomic analysis and defining the most relevant pathways involved in the *Ceratothoa* pathology.

## Materials and Methods

### Animals, Samplings, and Ethical Statement

European sea bass (*D. labrax*) fingerlings (average weight = 33.2 g ± SD 5.7, fork length = 14.5 cm ± SD 1.1) were sampled from a commercial aquaculture facility at the eastern Adriatic Sea coast (Croatia) during a vaccination campaign in mid-November 2016 (water temperature = 17–19°C). This developmental stage was chosen because fingerlings are the most susceptible age category to the isopod infection and consequentially face the highest mortality while already having a fully developed immune system. Buccal cavities of the fingerlings were checked for the presence of *C. oestroides* and the infected (INF, *n* = 10) and uninfected (CTRL, *n* =10) fish were euthanized by an overdose of the anesthetic, MS-222 (0.1 g/L), and immediately dissected. All infected fish harbored a single, matured female from *C. oestroides* and a matured male. Samples of tongue at the female attachment site, head kidney, liver, and spleen were removed from each animal and stored in RNAlater RNA Stabilization Reagent (Qiagen) until RNA isolation. The parasite was removed before storing tongue samples in RNAlater. Pieces of organs from equivalent areas were obtained from control uninfected fish.

Fish were sampled during a procedural health examination at the aquaculture facility performed by a veterinary service, and in accordance with the Guidelines of the European Union Council (Directive 2010/63/EU) and the Croatian legislation (Zakon o zaštiti životinja, NN 135/06 and 37/13; Pravilnik o zaštiti životinja koje se koriste u znanstvene svrhe, NN 55/13). The procedures have been approved by the Committee for Animal Welfare, Institute of Oceanography and Fisheries, Split, Croatia (IACUC, approval number 134/2). All efforts were made to minimize the suffering of the fish used for the study in accordance with the aforementioned National Legislation and the current European Union Legislation (86/609/EU).

### RNA Extraction

The pieces of organs were removed from the RNAlater, and ~50 mg were homogenized in 1 ml of TRI Reagent (Applied Biosystems) Solution from the MagMAX™-96 for Microarrays total RNA Isolation Kit (Applied Biosystems) and the RNA was subsequently isolated following the manufacturer's instructions. The RNA concentration was measured using a Nanodrop 2000C Spectrophotometer (Thermo Fisher Scientific). Quality and integrity of the isolated RNA were checked on an Agilent Bioanalyzer 2,100 total RNA Nano series II chip (Agilent); the RNA integrity number (RIN) values ranged between 6 and 9.

### Reverse Transcription and Gene Expression Analysis

A preliminary targeted PCR-array was performed on all samples of the 10 (control) CTRL and 10 infected (INF) fish to determine the best responders, the most relevant organs, and the best quality samples to be selected for Illumina RNA sequencing. Reverse transcription was performed using the High-Capacity cDNA Archive Kit (Applied Biosystems) from 500 ng of RNA following the manufacturer's instructions. Negative control reactions excluding the reverse transcriptase were performed.

A 96-well PCR-array layout was used for simultaneous profiling under uniform conditions of 23 selected genes. These targeted genes included genes involved in B cell responses (*IgM, IgT*, and *IgD*), T cells (*CD3*ζ, *CD4-1*, and *CD8*α), antigen presentation (*mhcI* and *mhcII*β), cytokines (*il6, il8, il1*β, *il4/13a1, il17a/f*, *il10*, and *tnf*α), starvation markers (*igf1, ghr1*, and *ghr2*), apoptosis markers (*cas3* and *cas6*), wound healing markers (*mmp9*), and iron transport and metabolism genes (*trf* and *frim*). The full names and primers of the selected genes can be found in Supplementary File 1A. Primers were designed to produce amplicons of 50–150 bp and tested so that all had similar efficiencies, which were always above 90% (R^2^ > 0.98). PCR reactions (20 μl) contained 3.3 ng of total input RNA, 4 μl of 5 × PyroTaq EvaGreen qPCR Mix Plus (Cultek Molecular Bioline), and specific primers at a final concentration of 0.45 μM. The PCR conditions consisted of an initial step at 95°C for 3 min, followed by 40 cycles of denaturation for 15 s at 95°C and annealing/extension for 60 s at 60°C. The specificity of the reactions was verified by visually analyzing the melting curves for each reaction performed. Fluorescence data acquired during the extension phase were normalized by the delta Ct method (18) using β*-actin* as a reference gene. This particular reference gene was chosen from several other candidates as the most stable among conditions in each sample using the GeNorm software. All individual β*-actin* Ct values were verified to have differences lower than 1 Ct value. Statistical differences (*P* < 0.05) between CTRL and INF samples for each primer and sample were determined by the Student's *t*-test or the Mann–Whitney–Wilcoxon test when normal conditions were not met. The same procedure was used to validate some immune-related differentially expressed (DE) genes found in the RNAseq study, and the information about the primers can be found in Supplementary File 1B.

### Illumina Sequencing

Using the preliminary PCR-array results, RNA samples of the tongue, spleen, and liver from six INF and six CTRL fish were selected for Illumina sequencing, yielding a total of 36 samples. Illumina RNA-seq libraries were prepared from 500 ng total RNA using the Illumina Tru-Seq^®^ Stranded mRNA Library Prep Kit (Illumina Inc.) following the manufacturer's instructions. All RNA-seq libraries (150–750 bp inserts) were sequenced on an Illumina NovaSeq 6,000 sequencer as 2 ×150 nucleotides paired-end reads according to the manufacturer's protocol. Image analysis and base calling were performed using the Illumina pipeline.

### Sample Quality Assessment and Bioinformatics Analysis

Approximately 615 million paired-end reads were obtained from the 36 sequenced samples with an average of 17 million reads per sample. FastQC v0.11.9 was used to perform quality analysis and libraries were filtered using PRINSEQ for quality >30 and <10% of Ns in the sequence. Filtered sequences were mapped using HISAT2 (19) and the reference European sea bass genome (dicLab v1.0c, http://seabass.mpipz.de). Cufflinks (20) was used to assemble the transcriptome for each sample and quantify the transcript expression in fragments per kilobase of transcript sequence per million base pairs sequenced (FPKM). These steps of the analysis were performed using the Galaxy web platform at usegalaxy.org (21). Non-annotated transcripts were subjected to a second round of annotation using the NCBI tool, blastx (22) with an *e-*value of 10e^−5^ as the cutoff threshold and the non-redundant (NR) database from NCBI. Before proceeding with the differential expression analysis, the Cufflinks data were quality checked using CummeRbund (23). An INF spleen sample that yielded an anomalous FPKM density distribution was removed from further analysis.

### Differential Expression Analyses, Statistics, and Visualizations

Differentially expressed transcripts between the INF and CTRL groups for each organ were calculated using the Bioconductor R package DESeq2 (24). The number of samples used in each comparison was *n* = 6 for INF and CTRL samples, except in the case of INF spleen samples, where *n* = 5. DE transcripts were considered at padj <0.05. Log2 fold change values were used to separate upregulated and downregulated transcripts. The lists of annotated total upregulated and downregulated genes in each organ were used to perform gene ontology (GO) analysis using the online tool, GOrilla (25), with a *p*-value threshold of 10e^−3^. Pathway analysis was performed using the ReactomePA R Bioconductor package (26, 27) that uses the hypergeometric model (28) to calculate *q*-values. It is to be noted that reactome and other available pathway analysis tools are databases based mainly on human genes and pathways and do not contain any information about the European sea bass. Hence, pathway analysis was performed after converting European sea bass identifiers into their human equivalents, whenever possible. We are aware that this information could be incomplete and does not take into account the different isoforms which are very common in fish genomes. However, this approach can still provide interesting hints that allow navigation through this massive amount of data in a more comprehensive way. Principal component analysis (PCA) and hierarchical clustering using the FPKM values for all DE transcripts were performed using the factoextra R package (29). The optimal number of clusters was determined using the gap statistic. The ellipse confidence level in the PCAs was set at 0.95.

## Results

### *Ceratothoa oestroides* Induces Significant Effects Not Only Locally but Also Systemically

**Figure 1:**
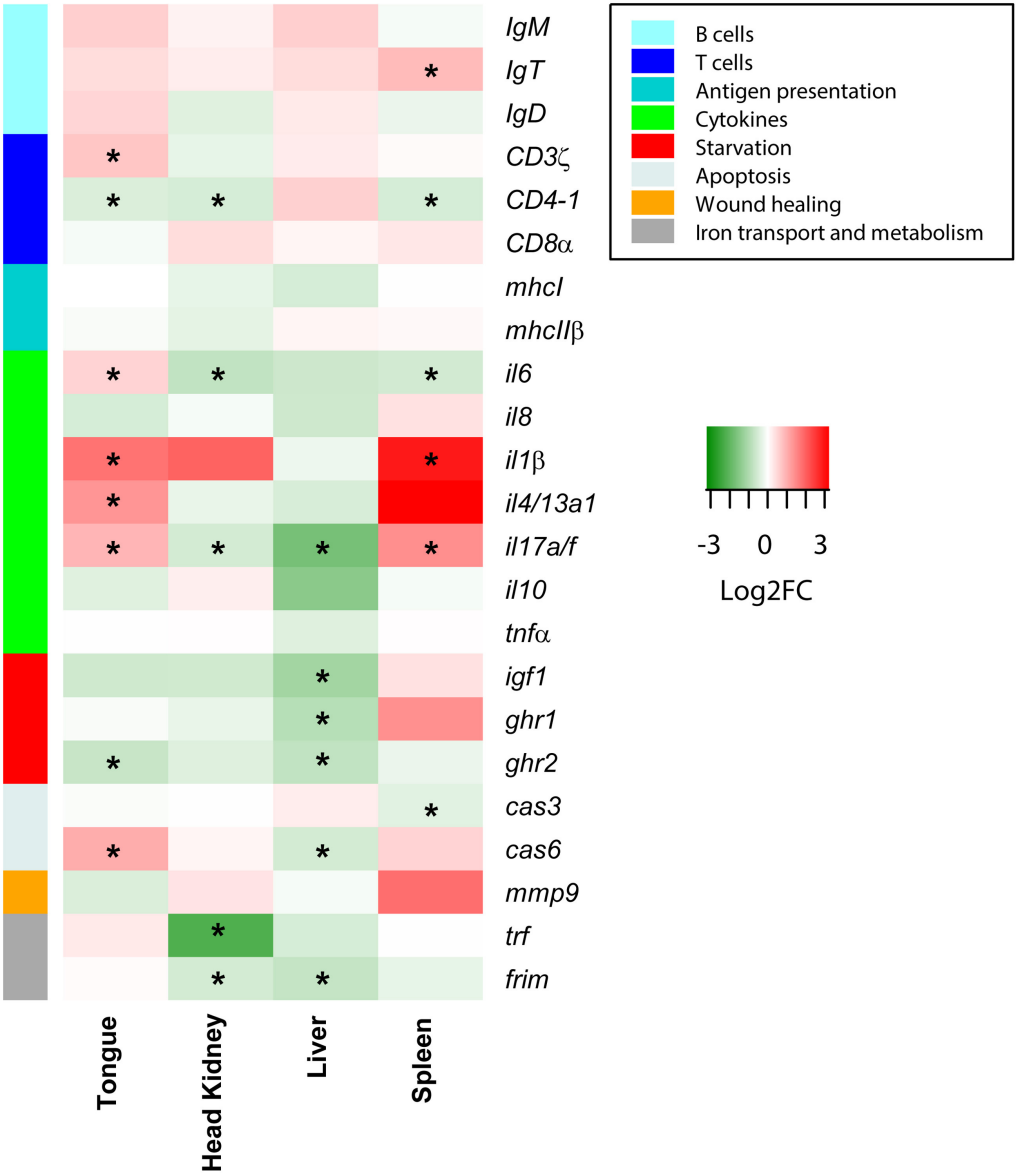
Heatmap depicting the PCR-array results of *Dicentrarchus labrax* samples from four organs. Red and green represent upregulated and downregulated genes, respectively, in *Ceratothoa*-infected fish relative to the uninfected controls. The color scale represents the Log2 fold change (Log2FC). Different colors on the left bar indicate the different pathways with which each gene was related. Asterisks represent statistically significant differences in the comparison of *n* = 10 infected and *n* = 10 control fish.

The preliminary PCR-array of 23 selected genes revealed that *C. oestroides* induced changes not only in the target organ, the tongue, but also in the liver, head kidney, and spleen. A strong B cell response was not detected; only a significant upregulation of *IgT* was found in the spleen of INF animals. Regarding T cells, although *CD3*ζ was significantly upregulated in the tongue, *CD4-1* was downregulated in the tongue, head kidney, and spleen. No changes were detected for *CD8*α. In the tongue of INF animals, the cytokines, *il6, il4/13a1, il1*β, and *il17a/f*, were significantly upregulated, while the latter two were also upregulated in the spleen. The effect on the starvation markers was evident in the liver, with significant downregulation of *igf1, ghr1*, and *ghr2* (also in the tongue) in INF animals. Iron transport and metabolism markers were downregulated in the head kidney, and the liver of the INF fish (Figure 1).

**Figure 2:**
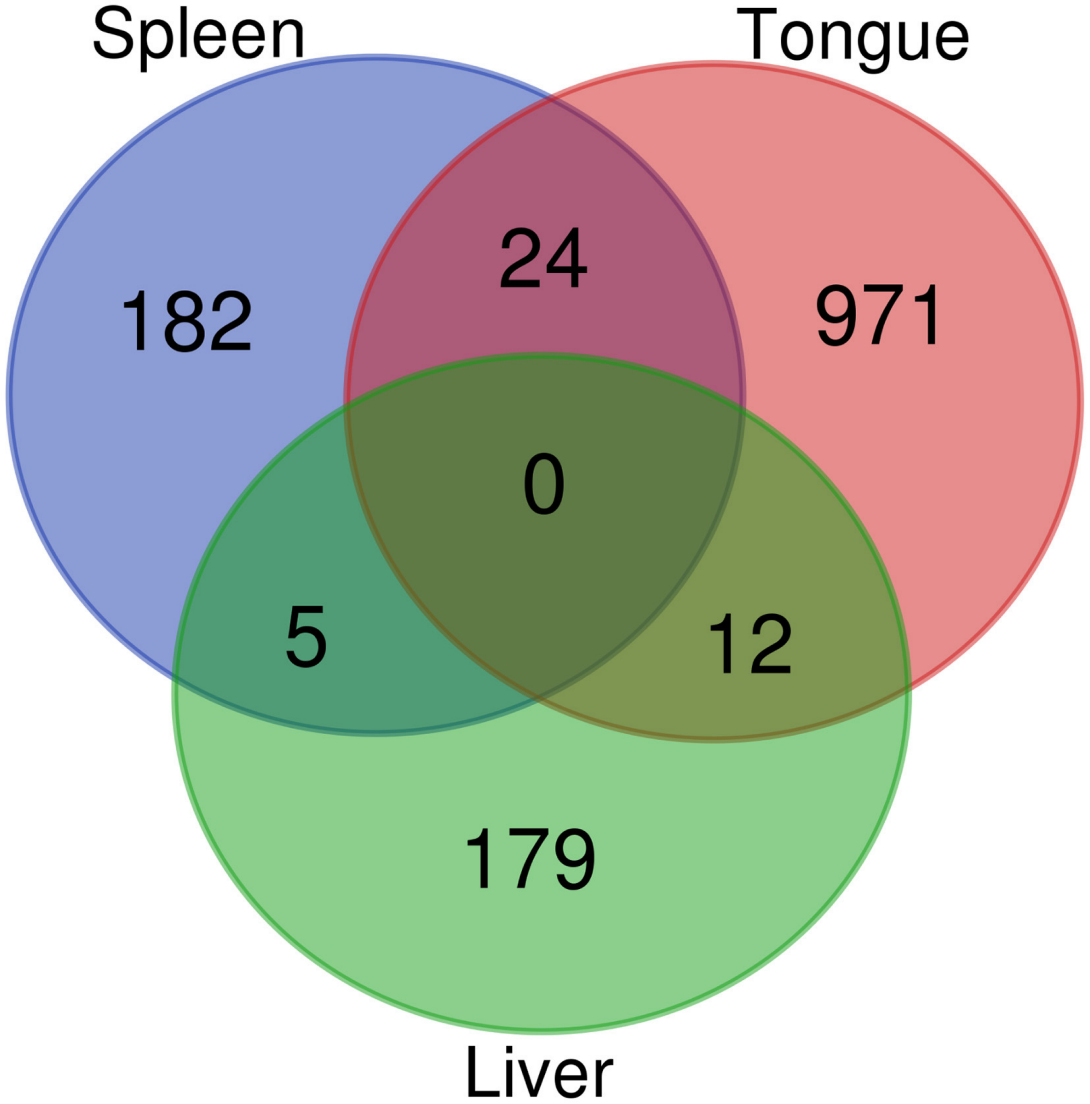
Venn diagram showing shared and unique differentially regulated transcripts among the three organs of *Dicentrarchus labrax* resulting from RNA sequencing (RNAseq).

RNA-sequencing of the tongue, liver, and spleen revealed that a total of 1,373 transcripts were DE upon infection with *C. oestroides*. As expected, the main affected organ was the tongue, with 1,007 differentially regulated transcripts, 597 upregulated transcripts, and 410 downregulated transcripts. The liver and spleen showed lower effects, with 196 (89 upregulated and 107 downregulated) and 211 (86 upregulated and 125 downregulated) DE transcripts, respectively. The Venn diagram of all DE transcripts revealed little overlapping, with no transcript commonly regulated among the three organs (Figure 2). Most of the DE transcripts were organ exclusive. Of interest are 24 transcripts that were commonly regulated in the tongue and spleen (Supplementary File 2). Among them, six are involved in immune responses (T cell receptor gamma chain C region, mannan-binding lectin serine protease 1-like (*masp1*), nuclear receptor subfamily 1 group D member 1-like, matrin-3, and two *IgHV* genes).

**Figure 3:**
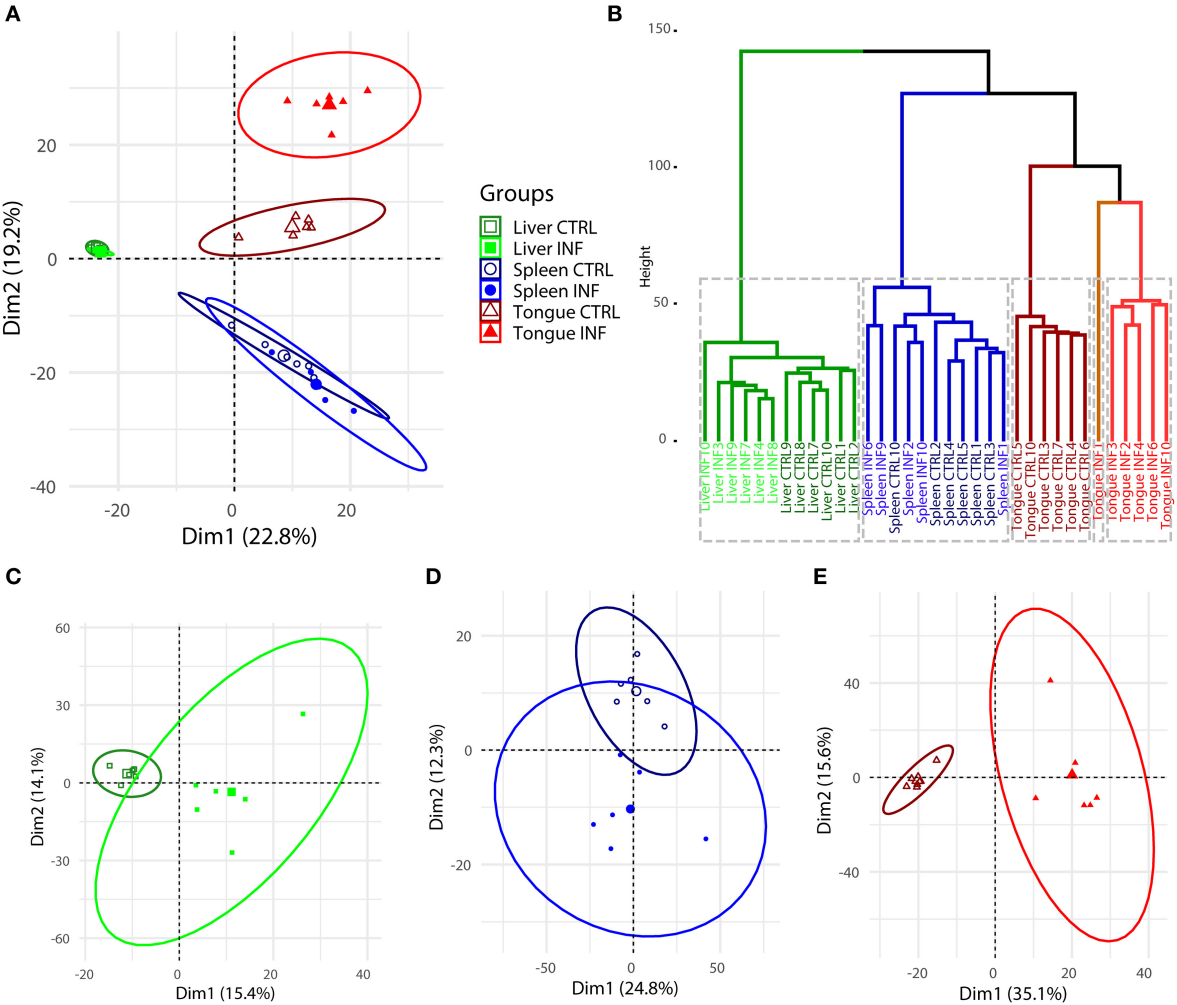
Principal component analysis **(A)** and hierarchical clustering **(B)** of all the individual samples and organs of *Dicentrarchus labrax* constructed based on the fragments per kilobase of transcript sequence per million (FPKM) values for each of the differentially regulated transcripts in *Ceratothoa*-infected (INF) vs. non-infected control (CTRL) fish. The optimal number of clusters in **(B)** was determined by the gap statistic (k = 5). Principal component analysis using all differentially expressed (DE) transcripts was also performed on the liver **(C)**, spleen **(D)**, and tongue **(E)** samples separately. The confidence level of the ellipses was 0.95 in all cases. The number of CTRL and INF samples was *n* = 6, except for INF spleen samples, where *n* = 5.

Principal component analysis (PCA) performed using the expression of the 1,373 DE transcripts showed a clear separation between the three organs studied (Figure 3A). As expected, the major effect of the infection was found in the tongue, whereas the separation of the CTRL and INF groups in the spleen and liver was not so evident in the overall PCA. Hierarchical cluster analysis endorsed the results of the PCA (Figure 3B). The optimal number of clusters, determined by the gap statistic, was *k* = 5, separating the CTRL from INF tongue samples in three clusters and the spleen and liver in the other two. Within the liver cluster, the separation between CTRL and INF animals was very clear. On the other hand, in the spleen, an INF sample grouped within the CTRL group and *vice versa*. PCA analyses showed in Figures 3C–E were constructed with the expression of the 1,373 DE transcripts, but for each organ separately, to allow a more detailed study without the differences among organs masking the differences between the INF and CTRL groups. Again the major differences, at the level of the number of DE transcripts and fold changes, occurred in the tongue (Figure 3E), followed by the liver (Figure 3C) and finally the spleen (Figure 3D). The CTRL and INF groups were separated by component 1 in the liver and tongue samples and by component 2 in the spleen samples.

### Functional Gene Enrichment Analysis

Out of the 1,373 DE transcripts, 1,299 (94.6%) were identified as known protein-coding genes and successfully annotated, including GO term assignment. Table 1 shows the number of enriched GO terms for each organ. The GO term analysis was performed by separating the upregulated and downregulated transcripts to create an overview on the direction of the changes. The complete list of the GO enrichment analysis and results is shown in Supplementary File 3. In the liver, enriched biological process ontologies include: response to hormone, ion transport, drug and carbohydrate metabolism, and mitochondrial organization. In the spleen, a significant enrichment of response to stimulus (stress), tissue development and organization, and immune response and inflammation was detected. In the tongue, among the many enriched ontologies, response to stimulus, *tissue repair and development*, and *muscle system processes* could be highlighted as the most abundant ontologies.

**Table 1:** Gene ontology (GO) terms significantly enriched in the different tissues.

**Tissue**	**GO level**	**Upregulated**	**Downregulated**
Liver	BP	1	10
	MF	0	6
	CC	3	7
	Total	4	23
Spleen	BP	17	7
	MF	1	0
	CC	5	2
	Total	23	9
Tongue	BP	79	121
	MF	29	10
	CC	31	11
	Total	139	142

*GO term enrichment (p-value cutoff 10e^−3^) was calculated by separating upregulated and downregulated transcripts per tissue. BP, biological process; MF, molecular function; CC, cellular component*.

### Pathway Analysis

**Figure 4:**
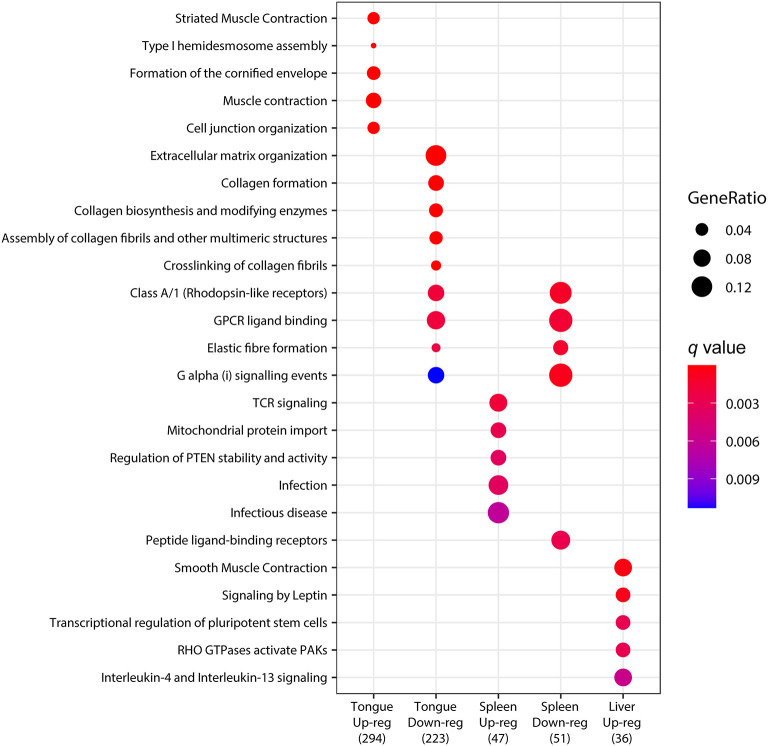
Dotplot pathway enrichment map showing the most significantly over-represented pathways for each set of upregulated or downregulated genes in each organ when comparing *Ceratothoa*-infected against CTRL *Dicentrarchus labrax* samples.

To determine statistically significant representations, the pathway analysis was performed using the genes that were significantly upregulated or downregulated (padj <0.05) upon infection in each organ. The results in Figure 4 summarize the most significantly regulated pathways. In the tongue, pathways related to muscle contraction and maintenance of tissue structure and integrity were found among the upregulated genes. However, extracellular matrix organization and collagen-related pathways were represented among the downregulated genes. G protein signaling events (Class A/1, GPCR ligand binding and G alpha (i) signaling events) appeared among the downregulated genes in the tongue and spleen, together with elastic fiber formation. In the spleen, several immune and inflammation-related pathways appeared among the upregulated genes. In the liver, no enriched pathway was found among the downregulated genes, but several pathways, including muscle contraction, lipid mobilization (signaling by leptin), and Il4/13 signaling, appeared among the upregulated genes. These results again highlight the minimal overlapping found among the organs, indicating an organ-specific response to the infection with *C. oestroides*.

### Relevant Gene Changes

All DE transcripts are depicted in Supplementary File 2. In an attempt to reduce and simplify the discussion, we selected several genes of interest for each organ associated with this particular disease-related category, also taking into account the results revealed by GO and pathway analysis.

**Figure 5:**
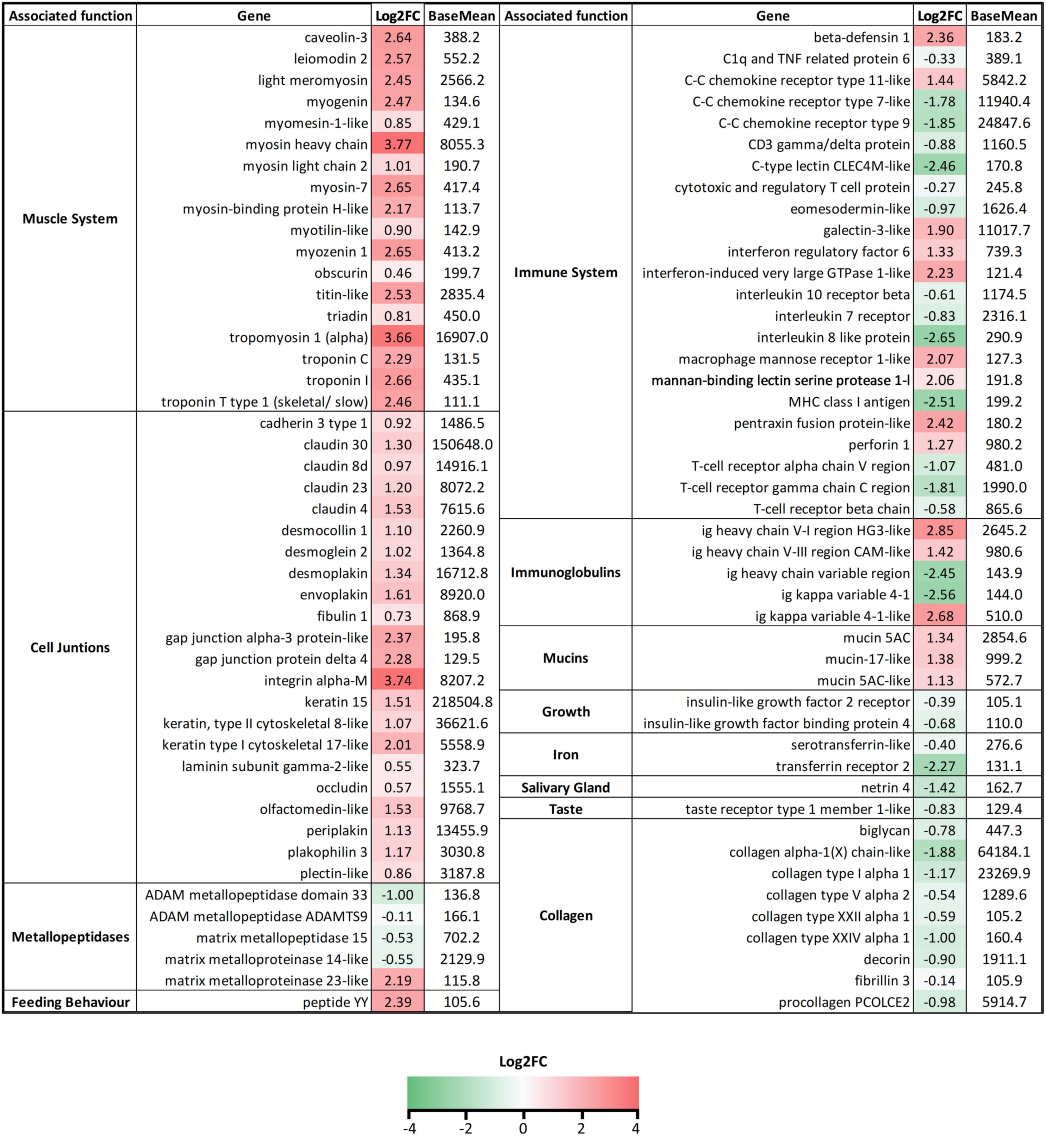
List of selected genes DE in tongue samples of *Ceratothoa*-infected *Dicentrarchus labrax*. The genes were grouped in categories regarding their associated function. The numbers represent the Log2 fold change values and the color scale corresponds to −4-0-4 (green-white-red) Log2FC. BaseMean values represent the average of normalized counts divided by the size factor, taken over all samples.

In the tongue (Figure 5), a large amount of genes related to muscle contraction and development and cell junctions were found to be always upregulated upon infection with *C. oestroides*. Metallopeptidases were mainly downregulated except for *mmp23*. Collagen and collagen-related proteins were all downregulated. Regarding immune-related genes, chemokines, T cell-related genes (cytotoxic and regulatory T cell protein, *CD3*γδ, *eomes*, and TCR genes) were downregulated, together with *mhcI, il8-*like, and *CLEC4M-*like lectin. Interferon regulated genes, perforin, pentraxins, macrophage mannose receptor 1, and defensins, were upregulated. A specific pattern was not found for immunoglobulins (Igs), while no constant chain was found regulated; variable regions from light and heavy chains were highly upregulated and downregulated, probably pointing toward a reshaping of the Ig repertoire upon infection. More than 15 Ig variable genes were found regulated in the tongue (Supplementary File 2). Growth, iron transport and metabolism, salivary gland, and taste-related genes were downregulated in the infected group. Peptide YY, related to feeding behavior, was highly upregulated in the infected fish.

**Figure 6:**
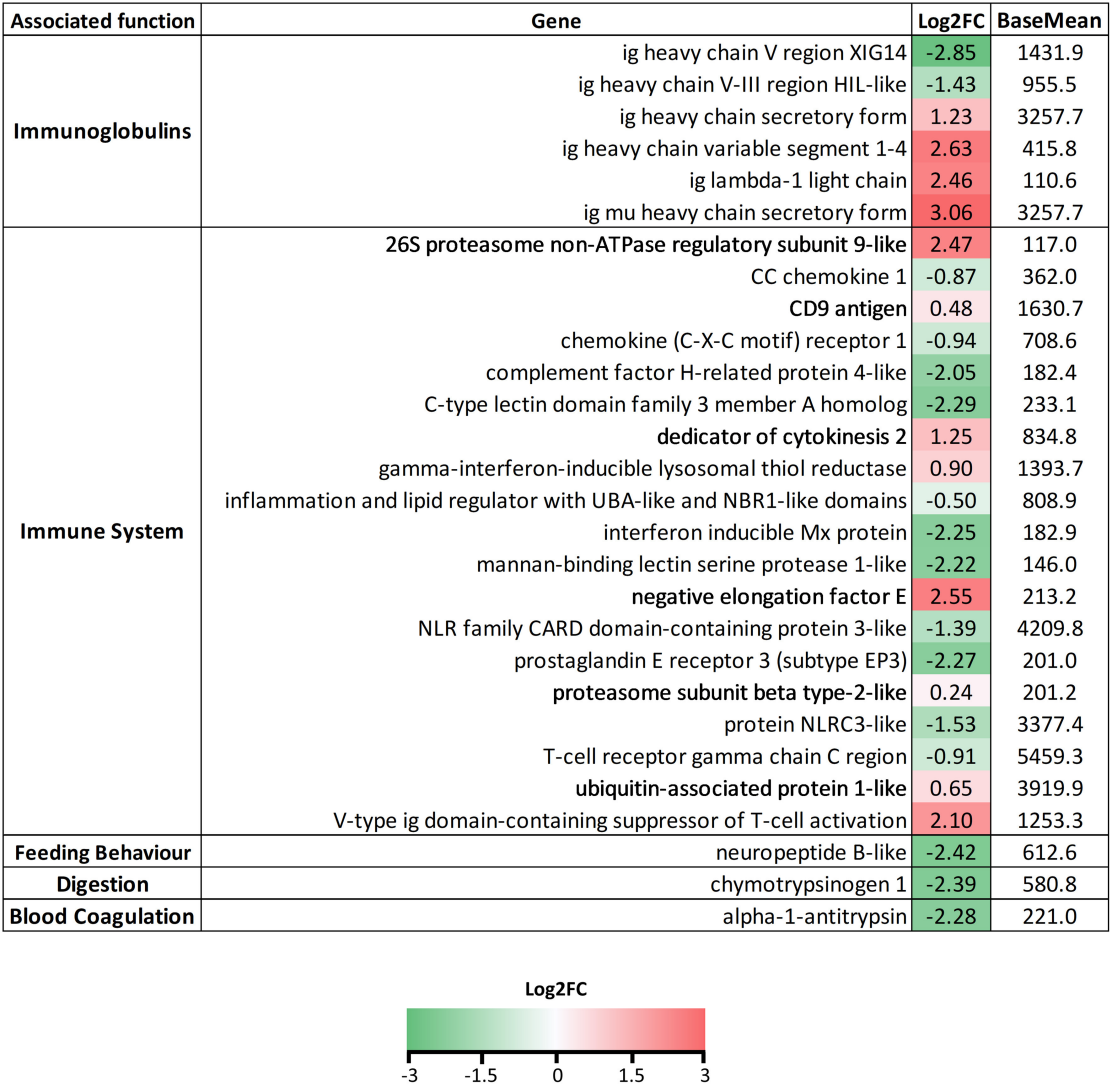
List of selected genes DE in the spleen samples of *Ceratothoa*-infected *Dicentrarchus labrax*. The genes were grouped in categories regarding their associated function. The numbers represent the Log2 fold change values and the color scale corresponds to −3-0-3 (green-white-red) Log2FC. BaseMean values represent the average of normalized counts divided by the size factor, taken over all samples.

In the spleen, we mainly focused on immune-related genes, as they were highly represented in the pathway analysis (Figure 6). Again, the variable segments of the Igs appeared upregulated or downregulated depending upon the gene. However, in the spleen we did find that some Ig constant genes (heavy and light chains) were significantly upregulated upon infection. Although several genes related to inflammation were upregulated, many immune genes appeared downregulated. Of interest, genes related to feeding behavior (*neuropeptide B-like*), digestion (*chymotrypsinogen 1*), and blood coagulation (*alpha-1-antitrypsin*) were highly downregulated in the infected animals.

**Figure 7:**
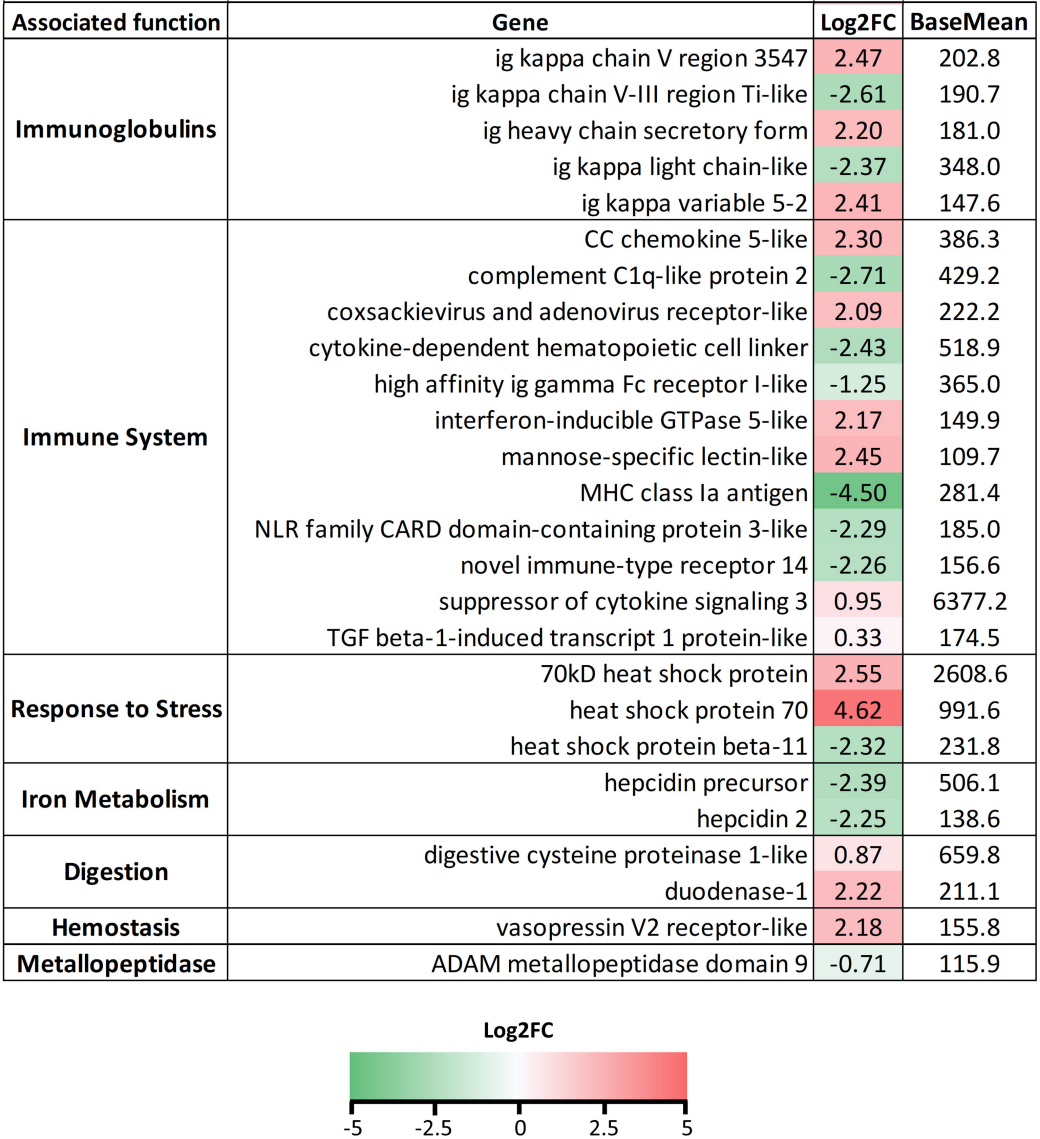
List of selected genes DE in liver samples of *Ceratothoa*-infected *Dicentrarchus labrax*. The genes were grouped in categories regarding their associated function. The numbers represent the Log2 fold change values and the color scale corresponds to −5-0-5 (green-white-red) Log2FC. BaseMean values represent the average of normalized counts divided by the size factor, taken over all samples.

Many immune-related genes were also found regulated in the liver of the infected fish (Figure 7). Constant Ig genes from heavy and light chains were found mainly upregulated, whereas a mixed profile was again found in the variable regions. Interferon regulated genes, mannose-specific lectin, some chemokines, and coxsackievirus and adenovirus receptor were upregulated, whereas complement proteins, NOD-like receptors, novel immune-type receptor 14, and cytokine-dependent hematopoietic cell linker were downregulated. The anti-inflammatory genes *socs3* and tgfβ-1-induced transcript 1 protein, were upregulated. Several transcripts annotated as *hsp70* or *hsp70-like* were found upregulated, whereas *hsp*β*11* was downregulated. Genes related to digestion and hemostasis were upregulated and the metallopeptidase *mmp9* was significantly downregulated.

Several genes detected as differentially regulated in the PCR-array were not found to be significantly regulated in the RNAseq analysis. This can be explained by the different number of samples used in each assay (*n* = 10 for the PCR-array and *n* = 6 for the RNAseq) and the different statistical methods and FDR corrections performed in the RNAseq, i.e., the latter using padj values as a cutoff value. Nonetheless, the expression trends were consistent between the two methods. For instance, *il1*β, the strongest upregulated gene in the tongue and spleen in the PCR array (Log2FC 1.8 and 2.9 in the tongue and spleen, respectively) was also upregulated in the RNAseq analysis (Log2FC 1.8 and 1.2), with padj > 0.05. The same happened with the starvation markers in the liver; *igh1, ghr1*, and *ghr2* had a Log2FC in the PCR-array of −1.2, −0.9, and −0.8, respectively, and a Log2FC of −1.5, −1.2, and −0.3 in the RNAseq (padj > 0.05). Supplementary File 4 shows the correlation between the results obtained from qPCR and RNAseq for genes of interest. Supplementary File 5 shows the results of the validation test for some immune-related genes, showing the correlation of the results obtained from the two techniques, fold changes, and *p*-values. The expression trends found for *masp1* (in the tongue and spleen), *ccr9* (in the tongue), *vista* (in the spleen), and *socs3* (in the liver) by qPCR were in agreement with those found in the RNAseq results.

## Discussion

The Cymothoidae family is one of the largest isopod families with approximately 40 genera and 380 species and is an example of how evolutionary drivers have induced a wide number of adaptations to a parasitic lifestyle. Among these adaptations, the attachment site depicts the versatility of the family, with genera that exclusively parasitize the gills, mouth (mouth-dwellers), external surfaces, or that reside inside the host flesh (flesh-burrowers) (30). In the past, this served as a base for drawing phylogenetic relationships within the family, as site-specificity is determined by the needs of the parasite and the limitations exerted by the morphology and the habits of the host (31). *Ceratothoa oestroides* is a mouth-dwelling cymothoid that induces limited pathology at the attachment/feeding site, mostly in the form of epithelium proliferation and a weak to moderate leukocyte infiltration (13). Given the extent of the damage inflicted in reared fry and fingerlings (6–10), in comparison to the restricted histopathology, we performed a transcriptomic analysis in the organs of fingerling sea bass to elucidate the mechanisms that potentially contribute to the elevated mortality in this class of fish.

As expected, the highest response in terms of activated transcripts was observed in the tongue and, to a lesser degree, in the spleen and liver. This suggests that, although there is a systemic response to *C. oestroides* parasitization, it is fairly weak compared to the local reaction (e.g., in the tongue) and also that it differs in type. Unexpectedly, the prevailing upregulated pathways in the tongue contributed to the activation of myocytes and cell–cell contacts. The enriched pathways *striated muscle contraction* and *muscle contraction* are processes that generate force through actin and myosin interaction and consequent ATP hydrolysis, which results in the movement of the tongue. Tongue muscles of the fish are employed to funnel the food farther into the mouth for processing and this intra-oral prey processing (chewing) seemed to occur more extensively than previously documented (32). The permanent positioning of *C. oestroides* on the surface of the tongue likely triggers this voluntary action with the purpose to mechanically detach the isopod. The superficial damage of the tongue-stratified squamous epithelium exerted by parasite chewing, pointed pereopods, ungulae, and pleopods continuous beating (13), induces sloughing of the uppermost cornified layer composed of the epithelia apoptotic cells. This process is supported by the upregulation of the pathways *formation of the cornified envelope, type I hemidesmosome assembly*, and *cell junction organization*. In mammalian skin and cutaneous mucosa of body openings, keratinocytes migrate upward from the basal lamina, undergo physiological and morphological changes to surface on the top, in the form of a translucent, cornified envelope (*stratum corneum*) composed of apoptotic cells that give structural stability, elasticity, mechanical resistance, and water repellence to the outside environment (33). In addition, hemidesmosomes, one of the five types of cell junctions, mediate adhesion of cells to the underlying basement membrane in the squamous epithelium and is highly dynamic, being able to disassemble quickly, i.e., during cell division, differentiation, or migration (34). The enriched pathways observed in the current study support the hypothesis that the local damage induced by the isopod is very mild and mostly superficial. Interestingly, Th2-polarized cytokine responses, which are usually observed in parasite infections (35), seem to be responsible for the imbalance in the cornified envelope, tight junction proteins, and skin β-defensins in humans with atopic dermatitis (36). It is intriguing to speculate whether *Ceratothoa* excretory/secondary products drive the local/systemic response of European sea bass toward anti-inflammatory pathways, subsequently also affecting the integrity of the *stratum corneum*, as a mechanical barrier. However, even though some elements of type 2 response were observed, with the upregulation of *il4/13a1* in the tongue and Il4/13 signaling pathway in the liver, there is no clear evidence of a specific type of response taking place. In fact, a general downregulation of T cell activity was quite evident in the tongue and spleen, conveying shared downregulation of T cell receptor gamma chain, and a T cell receptor alpha and beta chains downregulation at the site of infection. The impairment of T-lymphocyte responses is an evasion strategy well-described in mammalian protozoan parasites (37). The interaction of this crustacean parasite with the fish host T cell responses deserves further studies.

In contrast to elevated epithelium reorganization in the tongue, *collagen and elastic fiber production, crosslinking*, and *assembly in fibrils* are significantly suppressed. This suggests that fibrosis, as defined by overgrowth, hardening, and/or scarring of various tissues attributed to the excessive deposition of extracellular matrix components, including collagen (38), does not take place as a result of isopod parasitization. Fibrosis is the outcome of chronic inflammatory reactions to different stimuli, persistent infections, autoimmune reactions, allergic responses, chemical insults, radiation, and tissue injury; it is effectuated by activated myofibroblasts, among others, by pathogen-associated molecular patterns (PAMPS), cytokines (IL-13, IL-21, TGF-β1), chemokines (MCP-1, MIP-1β), angiogenic factors (VEGF), growth factors (PDGF), peroxisome proliferator-activated receptors (PPARs), acute phase proteins (SAP), caspases, and components of the renin–angiotensin–aldosterone system (ANG II) (38). Interestingly, whole-cell signal transduction in the tongue is silenced through the downregulation of the G-protein coupled receptors (GPCRs), class A/1 (rhodopsine-like receptors), and G alpha (i) signaling events. GPCRs (including rhodopsin class A) are pleiotropic receptors detecting a large range of extracellular signals (from photons to hormones and neurotransmitters), consequently triggering numerous intracellular transduction cascades that reflect on many physiological functions (39). It also regulates the activity of the G alpha (i), whose classical signaling mechanism consists of cAMP-dependent pathway inhibition through the inhibition of adenylate cyclase (40). Consecutively, a decreased production of cAMP results in the decreased activity of cAMP-dependent protein kinases (PKA). In contrast, during the infection with *C. oestroides*, the downregulated GPCRs and G alpha (i) might in turn prompt an increased activity of PKA. This kinase with a broad specificity can phosphorylate many substrates, mediating multiple protein–protein interactions, thus is involved in almost every major pathway in eukaryotic cells, including cell division, cell death, growth, differentiation, and memory (41). In the tongue of the infected sea bass, stimulated PKA might indeed help to sustain a parasite locally by stimulating cell proliferation, but since the same cascade was also silenced in the spleen, a plausible reason could be PKA involvement in histone phosphorylation, a process primarily associated with DNA damage response and transcription regulation, among the rest (42).

As expected, upregulated pathways in the spleen involved infection- and immunity-related tasks, such as the activation of signaling cascades that determine cell fate by regulating cytokine production, cell survival, proliferation, and differentiation (*T cell receptor pathway*); proteasome-mediated degradation (*regulation of PTEN stability and activity*); translocation of proteins from cytosol to mitochondria, where they are integrated into dynamic networks, bioenergetics, used for mitochondrial morphology and coupling to the endoplasmic reticulum (*mitochondrial protein import*); and pathways related to infection (*infection and infectious diseases*). Perturbation of the spleen pathways due to infection with *C. oestroides* shows that the primary local effect is disseminated systemically in the host. Interestingly, among the most upregulated immunity-related genes are the *negative elongation factor E*, known for its role in inducing the expression of anti-inflammatory genes (43), and *V-type Ig domain-containing suppressor of T cell activation (vista)*, which has been recently described to interact with galectin-9, consequently inducing apoptosis in cytotoxic T cells in humans (44). Although galectin-9 was not upregulated in the spleen, it was found significantly expressed in our samples (mean expression: 1123.9 normalized counts). In relation to the complement system, *masp1*, a key enzyme for the activation of the lectin complement pathway (45), was found significantly upregulated in the tongue and downregulated in the spleen. In contrast, a significant downregulation of *masp* found in the skin and liver of common carp infected with a ciliate protozoan parasite, *Ichthyophthirius multifiliis*, was proposed as a counter-measure of the parasite to avoid complement activation (46). The opposed expression levels found in the tongue, the attachment site of *C. oestroides*, and in the spleen, the main antigen-processing organ, could be attributed to surface bacteria colonizing host tissues and reactivating complement pathways in the former and to isopod-mediated suppression in the latter. Similarly, studies performed in bacterial infections in tongue sole (*Cynoglossus* sp.) and zebrafish showed an upregulation of this gene in different tissues (47), clearly suggesting that *masp1* expression differs among fish and infection models.

Lastly, the liver was not majorly affected by isopod parasitization, though the upregulation of a variety of seemingly unconnected pathways was detected. These include pathways engaged in smooth muscle contraction after a particular stimulus (autonomic nervous system, hormones, autocrine/paracrine agents, and local chemical signals); transcriptional regulation of pluripotent stem cells that epigenetically drives cellular pluripotency, differentiation, and self-renewal/proliferation in the liver (48); RHO GTPase-activated PAKs, which are serine/threonine kinases mainly implicated in cytoskeletal rearrangements (49); and interleukin-4 and−13 signaling. The latter belongs to the repertoire of T-helper 2 cell (Th2) cytokine production, known to be activated in protection against intestinal worm infection (50). More importantly, IL-4 and stimulated Th2 cells represent a potent B cell growth factor that promotes humoral immunity (51), which, together with the regulation of Ig genes, would imply that a titer of specific anti-*Ceratothoa* Igs could be produced systemically during the course of the infection. Previously, observed infiltration of IgM^+^ cells at the infection site could suggest the activation of a specific immune response to *C. oestroides*, but IgM production could be alternatively a response to bacteria present in the biofilm that usually covers the body parts of the isopod (13). The induction of a B cell response is supported by the regulation of Ig genes in all organs studied. Particularly, the strong upregulation and downregulation of many variable Ig genes may indicate that the sea bass Ig repertoire is being reshaped upon infection with *C. oestroides*. The possibility that part of the detected changes in Ig expression could be due to sterile transcription should not be discarded. However, the magnitude of the changes detected clearly indicates that parasitization affects the Ig response. To solve this question, high-throughput Ig sequencing should be conducted to study the Ig repertoire, the functional clonotypes produced, and the response of the different isotypes during this host-parasite interaction. Regretfully, the Ig locus of European sea bass is not completely characterized and the sequences for all V, D, and J genes have not been identified and annotated, appearing in the genome as predicted or similar sequences, as is the case for many fish genomes. However, Ig repertoire analysis has been applied in a few fish models (52, 53) including studies with metazoan parasites (54, 55). Accordingly, parasitic infections induced a dysregulation of the Ig response characterized by a polyclonal expansion of diverse Ig subsets in trout (54) and gilthead sea bream (55). This condition was interpreted as an immune-evasion strategy elicited by the parasites aiming to dilute parasite-specific antibodies. Whether a similar effect takes place in this host-parasite model still remains to be determined. Further efforts need to be addressed to define European sea bass Ig locus and the role of the expression of Ig subsets in the frame of the infection with *C. oestroides*. In addition, whether this repertoire reshaping is being induced directly by the parasite or indirectly by contact with bacteria remains to be studied.

The only direct evidence of the perturbation of host energy reserves in the pathway analysis is the liver upregulation of leptin signaling. Leptin is an adipokine that controls food intake and energy homeostasis. In humans, it is implicated in obesity-related diseases and also inflammation. Normally, the depletion of fat stores triggers a leptin decrease and an increase in appetite and a lower energy expenditure to restore the fat to original levels (56). In contrast, in the infected sea bass, leptin was upregulated, which might prevent further appetite and fat storage, resulting in decreased body weight. However, available data suggest that elevated leptin itself may attenuate downstream leptin action so that leptin functions primarily in defense against decreased body weight rather than in limiting increases in body weight (56). Chronic weight loss is a common clinical sign in isopod infections (6, 8–10), and perturbed leptin in liver gives a molecular support to the process. The inhibition of appetite is further supported by the strong upregulation of peptide YY at a local level, which in mammals has been shown to inhibit appetite and reduce obesity (57). Moreover, the downregulation of genes related to starvation and growth (*igf1, ghr1*, and *ghr2*) at the local and systemic levels also reflect nutrient deficiency and impaired growth. These genes have been established as key performance indicators of growth, aerobic scope, and nutritional condition in gilthead sea bream (58, 59). In fact, the intestinal parasite, *Enteromyxum leei*, which causes severe anorexia in gilthead sea bream, induced downregulation of these markers (60, 61). In European sea bass, infections with the ectoparasite *Amyloodinium ocellatum*, also resulted in a significant downregulation of genes related with growth (*igf1*) and appetite (*npy*) (62).

**Figure 8:**
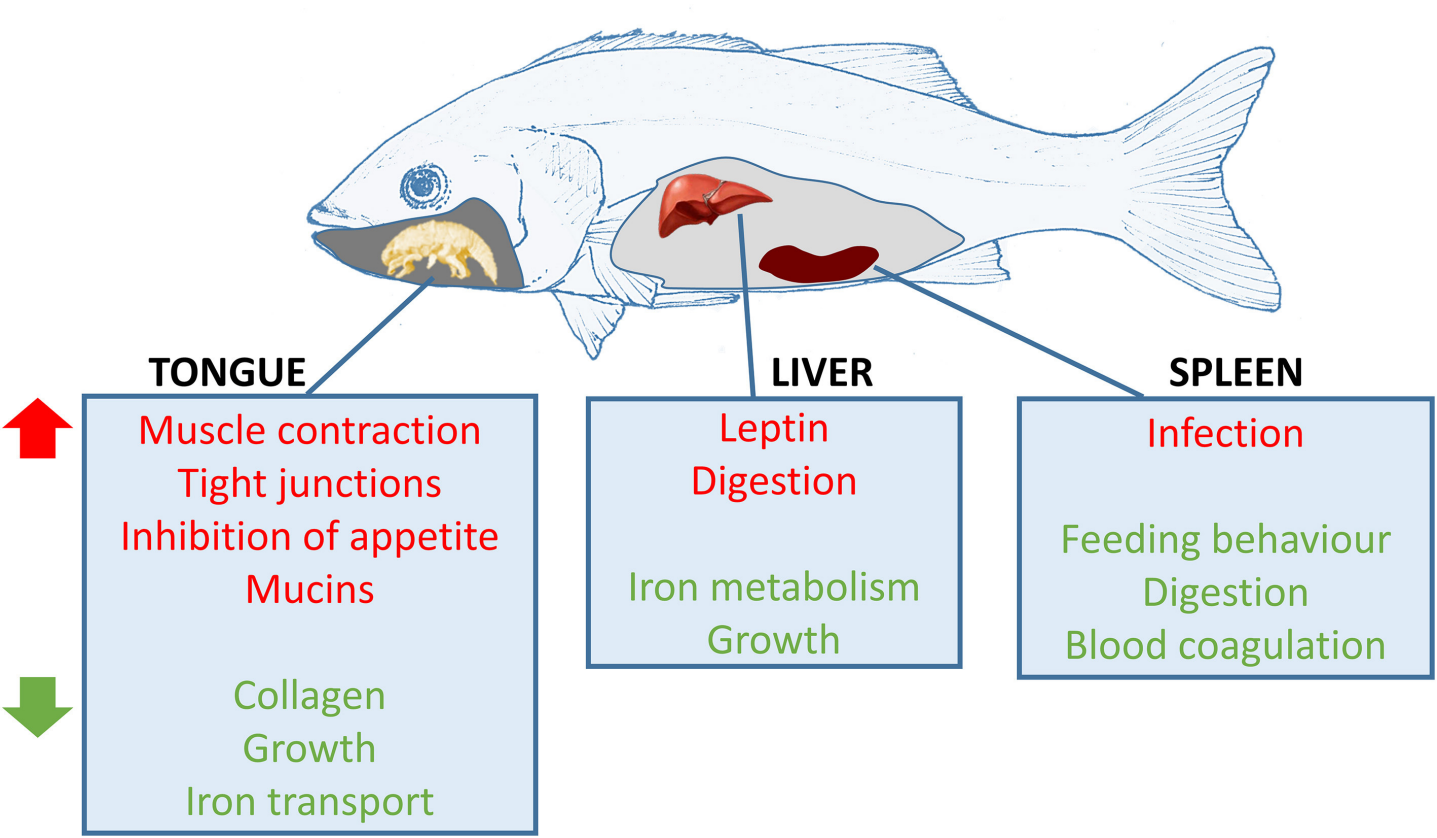
Schematic representation of the most affected pathways in European sea bass infected with *Ceratothoa oestroides*. Red pathways are upregulated and green pathways are downregulated in the infected animals. Fish drawing by Prof. M.L. Fioravanti (University of Bologna).

To conclude, infection with *C. oestroides* in European sea bass induced a very potent effect at the local level (the tongue) but also provoked important effects systemically. A schematic summary of these effects is shown in Figure 8. In the tongue, muscle contraction and cell junction pathways were upregulated, whereas collagen-related pathways and elastic fiber formation were downregulated. The host was able to mitigate the inflicted damage by successfully regenerating the tissue, instead of activating reparative processes (e.g., formation of connective tissue to repair epithelia). Spleen reaction was more addressed to an immune response against the parasite. However, there was no clear evidence of a specific type of response occurring during this infection, and only a general reshaping of the Ig response was observed. It is clear that the parasite is modulating adaptive (B and T cell responses) and innate (complement) parameters, in most cases downregulating or dysregulating the expression of genes in a strategy to overcome the host-immune defenses. The current results define important starting points to study parasite-mediated immune-evasion strategies in future studies. Several molecules related to growth deficiencies and starvation were regulated in the liver and tongue supporting the massive weight loss suffered by the infected fish. To our knowledge, this is the first transcriptomic study of fish infected with a mouth-dwelling parasite, and the results define key mechanisms involved in this mucosal tissue response. Future studies should be conducted in order to better define the observed Ig response and the immune pathways in order to find solutions to this infection in aquaculture.

## Data Availability Statement

The datasets generated in the RNA sequencing can be found in the Sequence Read Archive (SRA) of NCBI under the Bioproject accession number PRJNA686786 (BioSample accession numbers: SAMN17124849 - SAMN17124884).

## Ethics Statement

The animal study was reviewed and approved by the Committee for Animal Welfare, Institute of Oceanography and Fisheries, Split, Croatia (IACUC, approval number 134/2).

## Author Contributions

AS-B and IM conceived the study. JH performed the fish sampling. MCP performed RNA extraction, molecular analyses, and performed the bioinformatics and statistical analyses. RD and ES prepared the libraries and performed the RNAseq. AS-B, IM, and MCP performed data interpretation and wrote the original manuscript. AS-B, IM, and RD performed funding acquisition. All authors read and edited the manuscript.

## Conflict of Interest

RD and ES are employed by the company Future Genomics Technologies. The remaining authors declare that the research was conducted in the absence of any commercial or financial relationships that could be construed as a potential conflict of interest.

## Supplementary Material

Supplementary File 1 Primers used in the PCR-array of *Dicentrarchus labrax* **(A)** and in the validation study **(B)**.

Supplementary File 2 Complete lists of all differentially expressed (DE) transcripts found in the study. Each tab corresponds to the DE transcripts found in each organ when comparing *Ceratothoa*-infected samples with control samples of *Dicentrarchus labrax*. Log2 fold change (Log2FC), padj values, and the related gene ontology (GO) categories are provided for each gene. In the spleen and liver tabs, bold red genes represent the 24 common regulated transcripts in these two tissues.

Supplementary File 3 GO enrichment analysis of DE genes in the tongue, spleen, and liver of *Ceratothoa* infected *Dicentrarchus labrax*. The tabs entitled “ORGAN EnrichGO” contain the detailed lists of enriched GO terms among upregulated and downregulated genes for each organ. The results are sorted by ontologies: biological process (BP), molecular function (MF), and cellular compartment (CC).

Supplementary File 4 Scatter plots showing the correlation between the qPCR results (expression values relative to β-*actin*) and the results obtained from the RNA sequencing (RNAseq) analysis (Counts) of different genes in the tongue, spleen, and liver of healthy (blue dots) and *Ceratothoa oestroides*-infected (red dots) European sea bass.

Supplementary File 5 Validation of RNAseq by qPCR. Scatter plots showing the correlation between the qPCR results (expression values relative to β-*actin*) and the results obtained from the RNAseq analysis (counts) of selected immune-related genes in the tongue, spleen, and liver of healthy (blue dots) and *Ceratothoa oestroides*-infected (red dots) European sea bass. The Log2FC and *p*-values are shown for each gene, tissue, and technique.
