# Supplementary material for: Ceratothoa oestroides Infection in European Sea Bass: Revealing a Long Misunderstood Relationship
Source: Front Immunol. 2021 Mar 11;12:645607. doi: 10.3389/fimmu.2021.645607 (PMC7991915; doi:10.3389/fimmu.2021.645607)
Supplement: Supplementary File 1 — Primers used in the PCR-array of Dicentrarchus labrax (A) and in the validation study (B). [file Data_Sheet_1.PDF]

Supplementary file 1: Primers used in the PCR-array (A) and in the validation study (B).

A

Name	Symbol	Accession number (GenBank)	F/R primers (5'-3')
Immunoglobulin M	*IgM*	KY173354	CGCTACTGCTTTGTGGAACA/ GCAAGTCAGGGTCACCATTT
Immunoglobulin T	*IgT*	KP096356	TGAGCTGCACAGATGAAGAGGATGAGTT/ GGAATATGAAGGAGGAGGCTGTGGACC
Immunoglobulin D	*IgD*	KU132360	TGACAGCCAAGACTGAGGTG/ AGGTCACCTTGGCATCAAAC
T-cell surface glycoprotein CD3 zeta chain	*CD3ζ*	KM225783	CTGATGCGTCTGAAGAGAATGGAGGC/ GTTCAAGCACCTGGTAAGGATCAGCATC
Cluster of differentiation 4-1	*CD4-1*	AM849812	GGGACATTGAGGGAGGAAAGTGGGAAT/ AGAGGGAGAAGAGCATCTGTGGAGCATTA
Cluster of differentiation 8 alpha chain	*CD8α*	AJ846849	AGTGCCCACCATCAAACCAACTCTATGC/ CCTTCTTGTTACACACACATGGCGTGGTAG
MHC class I antigen	*mhcI*	AM943118	ACATAGCATTTGACCTGAGGACAGAGACAT/ CCTGTGGCACTGGAGCGACC
MHC class II antigen beta chain	*mhcIIβ*	AM113466	GCCAGCCTGAGAGAACCT/ GCTCCGATGGCGATCTTGT
Tumor necrosis factor alpha	*tnfα*	DQ070246	TCTACAGCCAGGCGTCGTTCAG/ CCGCACTTTCCTCTTCACCATCGT
Interleukin 1 beta	*il1β*	AJ311925	CATGAGCGAGATGTGGAGATCCAAGAT/ CATTGTCAGTGGGTGGTGGGTAATC
Interleukin 6	*il6*	AM490062	CATGCCCTGAGAAGTCCA/ TTGAGAAGAGCTGTGTAAGTGA
Interleukin 8	*il8*	KM225777	CAATCAGCAGGGACTACAACACACA/ CTGTCTGGAGGGATGATCCTTGACT
Interleukin 10	*il10*	DQ821114	CAGTGCTGTCGTTTTGTGGAGGGTTTC/ TCTCTGTGAAGTCTGCTCTGAGTTGCCTTA
Interleukin 4/13 a 1	*il4/13a1*	KJ818332	GCGTGAACACGAAGAATTGA/ GACGTCTGAAGGGACCACAT
Interleukin 17 a/f	*il17a/f*	KJ818335	GTTGTTTGCTGAACGGCTGTCTC/ CTCCAGGTTCGGGTCCTCTCT
Caspase 6	*cas6*	AM988220	ACAAGTGCAACAGCCTTGTG/ CAGCTCACTGTCCACAGCAT
Caspase 3	*cas3*	DQ345774	CTGATTTGGATCCAGGCATT/ CGGTCGTAGTGTTCCTCCAT
Ferritin middle subunit	*frim*	FN908856	CCTGGAGACCCACTACCTGA/ CAGGTACTCCGCCATCTTGT
Transferrin	*trf*	FJ197144	ACCGTTGATTGCCGGAATGCC/ ACTGCCATTGCGTCAGCCTCTT
Insulin like growth factor I	*igfI*	AY800248	TAGCCACACCCTCTCACTACTG/ CCTGTTGCCGTCGGAGTC
Growth hormone receptor type I	*ghrI*	AF438177	GGTGGATGCTGAGGATGC/ GGTGTCTGAGCCCTGGTT
Growth hormone receptor type II	*ghrII*	AY642116	TCCAGTCCAGAGCCCTAC/ ACGACCTCACCTCACTCA
Matrix metallopeptidase 9	*mmp9*	FN908863	CCTTCGTGTTCCTGGGTAAA/ AGGACAAAAGCCCCATTTCT
Beta actin	*actβ*	AY148350	TCCTGCGGAATCCACGAGA/ AACGTCGCACTTCATGATGCT

B

Name	Symbol	Identifier*	F/R primers (5'-3')
Mannan-binding lectin serine protease 1-like	*masp1*	LG22-25_257477	ACATCCAGGTTTTGGCAGAG/ TCGGGGTGCTTTCATAGTTC
C-C chemokine receptor type 9	*ccr9*	LG18-21_201066	ACAGGGCCAACTGAAAACAC/ GTGGCGAACAGTGCTGTAGA
V-type Ig domain-containing suppressor of T-cell activation	*vista*	LG11_85813	AGTGACGGGGAACTGTCAAC/ GGACATCTCGCCTTTAGCTG
Suppressor of cytokine signaling 3	*socs3*	LG8_281455	GCCCCTCTCGACTTCTCTCT/ AGCGTCGTACTCCTCCAGAA

*Transcript identifier in the European sea bass genome (http://seabass.mpipz.mpg.de/)
