# Supplementary material for: Ceratothoa oestroides Infection in European Sea Bass: Revealing a Long Misunderstood Relationship
Source: Front Immunol. 2021 Mar 11;12:645607. doi: 10.3389/fimmu.2021.645607 (PMC7991915; doi:10.3389/fimmu.2021.645607)

Supplementary file 4: Scatter plots showing the correlation between the qPCR results (expression values relative to *β-actin*) and the results obtained from the RNAseq analysis (Counts) of different genes in tongue, spleen and liver of healthy (blue dots) and *Ceratothoa oestroides* infected (red dots) European sea bass.

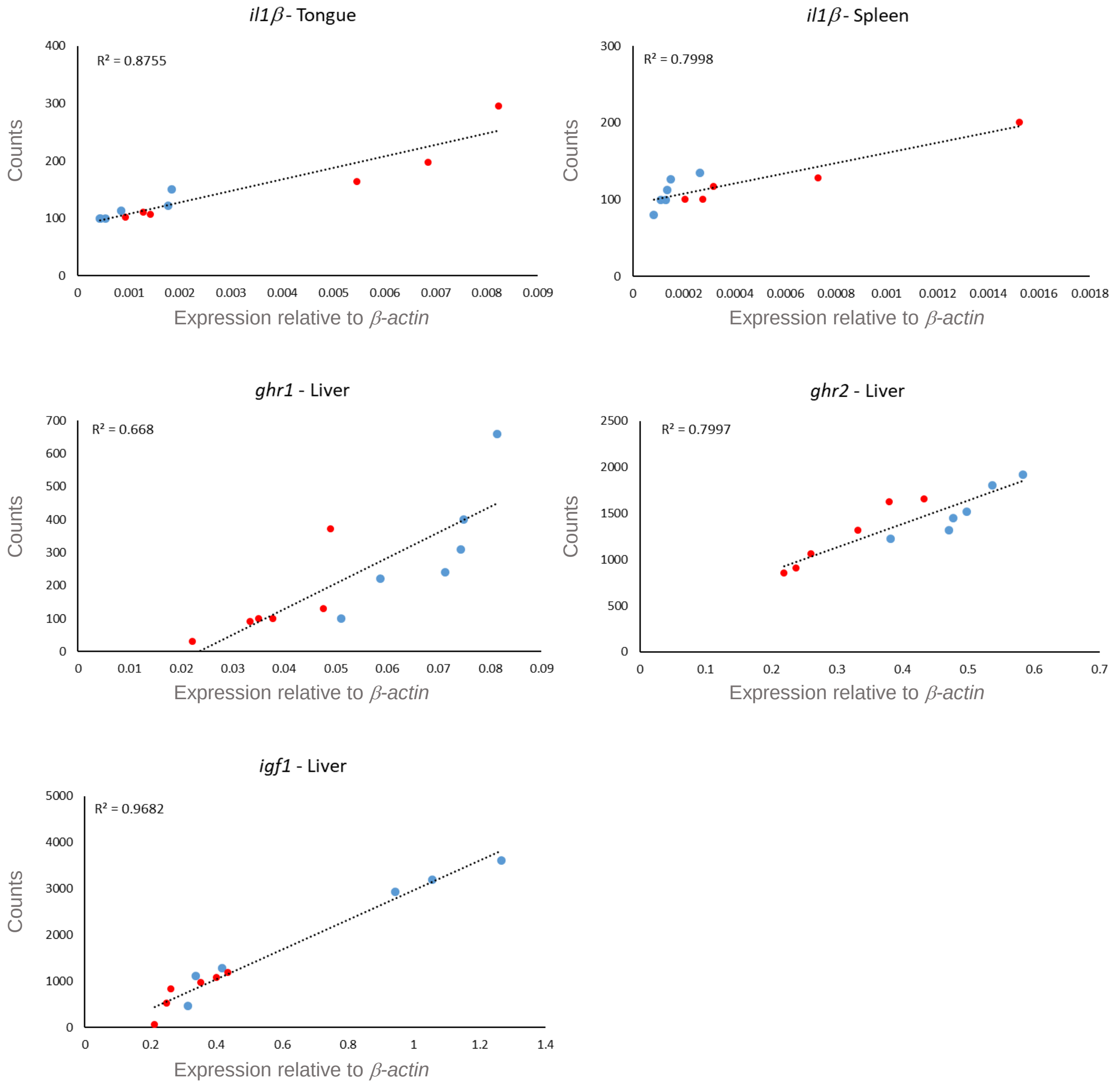

Supplement: Supplementary File 4 — Scatter plots showing the correlation between the qPCR results (expression values relative to β-actin) and the results obtained from the RNA sequencing (RNAseq) analysis (Counts) of different genes in the tongue, spleen, and liver of healthy (blue dots) and Ceratothoa oestroides-infected (red dots) European sea bass. [file Data_Sheet_4.pdf]
