# Supplementary material for: Ceratothoa oestroides Infection in European Sea Bass: Revealing a Long Misunderstood Relationship
Source: Front Immunol. 2021 Mar 11;12:645607. doi: 10.3389/fimmu.2021.645607 (PMC7991915; doi:10.3389/fimmu.2021.645607)

Supplementary file 5: Validation of RNAseq by qPCR. Scatter plots showing the correlation between the qPCR results (expression values relative to *β-actin*) and the results obtained from the RNAseq analysis (Counts) of selected immune related genes in tongue, spleen and liver of healthy (blue dots) and *Ceratothoa oestroides* infected (red dots) European sea bass. The log2 fold change (log2FC) and *P* values are shown for each gene, tissue and technique.

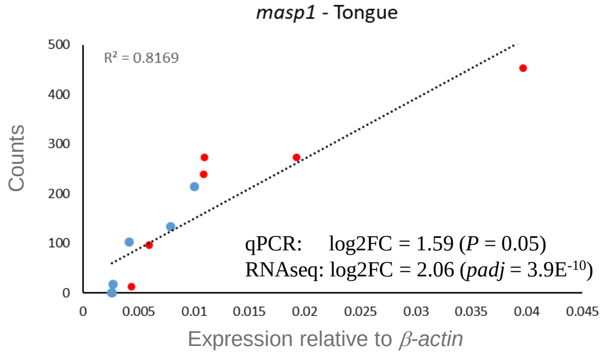

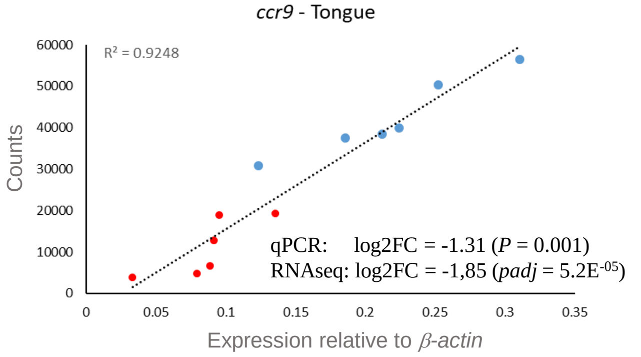

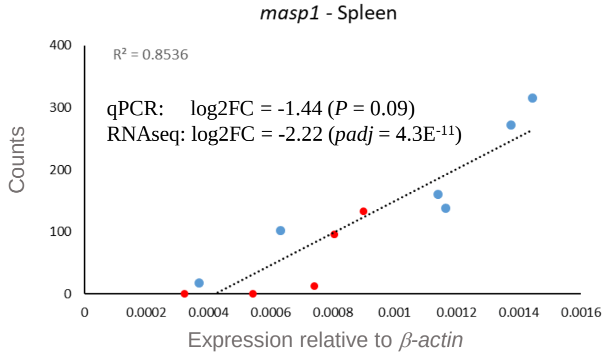

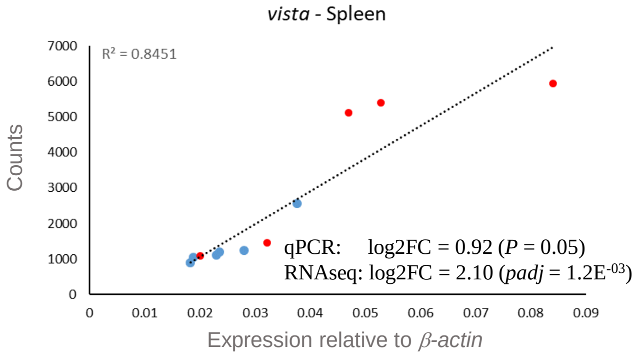

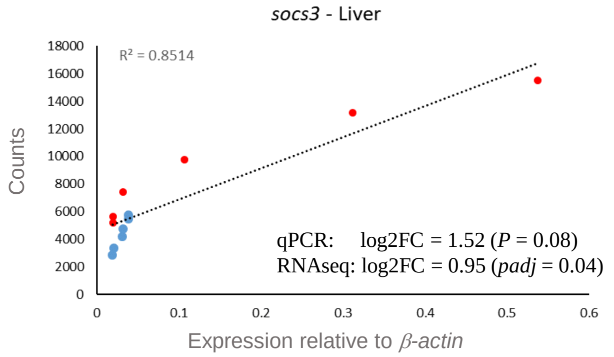

Supplement: Supplementary File 5 — Validation of RNAseq by qPCR. Scatter plots showing the correlation between the qPCR results (expression values relative to β-actin) and the results obtained from the RNAseq analysis (counts) of selected immune-related genes in the tongue, spleen, and liver of healthy (blue dots) and Ceratothoa oestroides-infected (red dots) European sea bass. The Log2FC and p-values are shown for each gene, tissue, and technique. [file Data_Sheet_5.pdf]
